# PseudoVelo: Inferring Gene Expression Derivatives Along Pseudotime as Pseudo-Velocity

**DOI:** 10.3390/ijms27146420

**Published:** 2026-07-19

**Authors:** Xinyuan Zang, Xin Shu, Zhen Zhou, Xiaoyong Wang, Jin Wang

**Affiliations:** 1 Nanjing Drum Tower Hospital Center of Molecular Diagnostic and Therapy, State Key Laboratory of Pharmaceutical Biotechnology, Jiangsu Engineering Research Center for MicroRNA Biology and Bio-technology, NJU Advanced Institute of Life Sciences (NAILS), School of Life Sciences, Nanjing University, Nanjing 210023, China; 2 Research Unit of Extracellular RNA, Chinese Academy of Medical Sciences, Nanjing 210023, China

**Keywords:** pseudotime, RNA velocity, differentiation, embryo, cellular process, ontogenesis, single-cell RNA sequencing

## Abstract

Understanding single-cell transcriptional dynamics during cellular differentiation and transition is fundamental to developmental biology. RNA velocity serves as a valuable approach for inferring these dynamics but is constrained by its reliance on simplified splicing kinetics. As a kinetics-free alternative, pseudotime-based approaches have been developed to reconstruct cellular transitions. Nevertheless, these approaches merely estimate cell–cell transitions by biasing the edges of a nearest-neighbor graph toward mature cell states. Here, we present PseudoVelo, a computational method that infers gene expression derivatives along pseudotime as “pseudo-velocity”. Utilizing the Generalized Additive Models commonly applied in pseudotime analysis, PseudoVelo fits the expression of each gene as a function of pseudotime. Then, by employing a central difference approximation, our method directly calculates the derivative of gene expression with respect to pseudotime, thereby obtaining the pseudo-velocity for each individual cell. Evaluated on multiple developmental processes, PseudoVelo demonstrates strong performance compared to CellRank 2, effectively recovering correct cellular trajectories using diverse temporal priors and demonstrating high resilience against various data perturbations.

## 1. Introduction

Understanding the complex transcriptional dynamics underlying cellular differentiation and lineage commitment is a fundamental pursuit in developmental biology. RNA velocity has emerged as a valuable approach for inferring the future state of individual cells during developmental processes [1,2,3,4,5]. However, current RNA velocity methods heavily rely on simplified models of splicing kinetics, which can produce conflicting trajectories in challenging settings [6,7]. As a kinetics-free alternative, pseudotime-based approaches have been developed to reconstruct cellular transitions. A prominent example is CellRank 2, which offers a straightforward approach based on the conceptual framework of Palantir to infer cellular dynamics by biasing a nearest-neighbor graph constructed from transcriptional similarity toward increasing pseudotime [8,9]. However, a shortcoming of this approach is that nearest-neighbor graphs are constructed based on gene expression resemblance, which does not necessarily reflect the actual transition probabilities between cellular states, raising concerns about their reliability.

We present PseudoVelo, a computational method that infers gene expression derivatives along pseudotime as “pseudo-velocity”, implemented as an accessible Python package. Our approach is built upon Generalized Additive Models (GAMs), a class of highly flexible statistical models designed to capture complex, non-linear relationships between variables [10]. In pseudotime analysis, GAMs are widely utilized because they excel at capturing the complex expression patterns of genes along a developmental continuum [11,12,13]. PseudoVelo utilizes GAMs to fit the expression of each gene as a smooth function of pseudotime. Then, by employing a central difference approximation on the fitted curves, our method directly calculates the derivative of gene expression with respect to pseudotime, thereby obtaining the pseudo-velocity for each individual cell.

To evaluate its performance, we applied PseudoVelo to multiple developmental datasets, including mouse erythrocyte maturation, zebrafish early embryogenesis, mouse pancreatic endocrinogenesis, and human hematopoiesis [9,14,15,16]. To this end, we introduced a quantitative benchmarking framework encompassing Cross-Boundary Direction Correctness (CBDC), Local Projection Consistency (LPC), and Initial/Terminal State Accuracy (ITSA). Our results demonstrate that PseudoVelo consistently outperforms CellRank 2, the leading alternative method in this domain, across these metrics. Furthermore, PseudoVelo exhibits robustness to the choice of input pseudotime; when provided with different temporal priors—such as Diffusion Pseudotime [17,18] or CytoTRACE scores [19]—as inputs, our method effectively recovers the expected developmental trajectories. Finally, we establish PseudoVelo’s high resilience to various data perturbations (such as unsupervised re-clustering, cell subsampling, and gene subsampling) and demonstrate its responsive reflection of erroneous temporal priors, cementing it as an effective tool for inferring cellular dynamics in development.

## 2. Results

### 2.1. The Design of PseudoVelo: Fitting Gene Expression Dynamics and Inferring Derivatives via Numerical Differentiation

We developed PseudoVelo, a computational framework that directly infers pseudo-velocity from pseudotime. The core principle of PseudoVelo is to establish a continuous statistical mapping between pseudotime and gene expression and subsequently utilize numerical differentiation to estimate the instantaneous rate of change for each gene.

**Figure 1:**
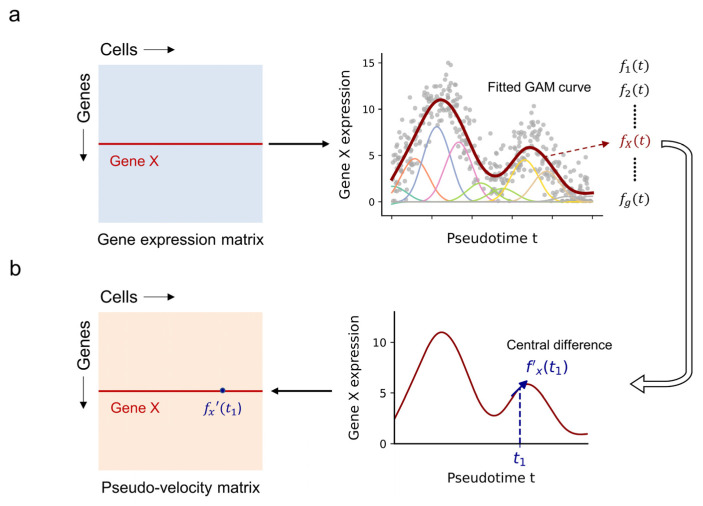
Overview of the PseudoVelo. (**a**) GAM fitting of gene expression dynamics. (**Left**) Schematic representation of the gene expression matrix, where the red line highlights a specific gene, Gene X. (**Right**) The expression of Gene X is fitted as a function of pseudotime t. Serving as a representative example, all genes are individually fitted as smooth functions of pseudotime, from $f_{1}\left(t\right)$ to $f_{g}\left(t\right)$. (**b**) Estimation of pseudo-velocity via central difference approximation. (**Right**) Based on the fitted curve of Gene X from (**a**), a central difference approximation is employed to estimate the time derivative of Gene X at a specific pseudotime $t_{1}$. (**Left**) By applying this approach, the pseudotime derivatives of all genes at the corresponding time points for all individual cells are calculated, yielding the pseudo-velocity matrix as the final output of the model.

The PseudoVelo pipeline begins by modeling the expression dynamics of individual genes along a pseudotime continuum (Figure 1a). We employ Generalized Additive Models (GAMs) equipped with a log link function and B-spline basis functions to non-linearly approximate the mean expression of each gene as a smooth function of pseudotime. PseudoVelo implements an adaptive knot allocation mechanism that dynamically adjusts the freedom of the fitted curve based on the effective time span of the data. When cell lineage or cluster labels are available, this adaptive fitting mechanism is applied at the subpopulation level to prevent over-smoothing in some clusters while capturing complex dynamics in main trajectories.

Once the continuous mean expression function is established for each gene, PseudoVelo calculates the pseudo-velocity for each individual cell (Figure 1b). In actual implementation, we employ a central difference approximation scheme rather than direct differentiation. By introducing a strictly controlled, sufficiently small time perturbation tied to the global time span of the manifold, the algorithm computes the time derivative of the expected expression value. Through this approach, the time derivatives of all genes at their respective pseudotime coordinates are calculated for every cell, ultimately yielding a pseudo-velocity matrix that can be seamlessly integrated into existing downstream analyses designed for traditional RNA velocity.

To quantitatively evaluate the performance of PseudoVelo and other methods, we designed a benchmarking framework encompassing three key metrics (see Methods for mathematical details). Because methods like CellRank 2 directly output a cell-to-cell transition probability matrix rather than velocities, and standard downstream analyses of traditional velocities also require their conversion into transition probabilities, all metrics are evaluated at the transition probability level to ensure a fair comparison. First, **Cross-Boundary Direction Correctness (CBDC)** assesses whether the inferred transition probabilities accurately capture the expected developmental directions between known sequential cell clusters. Second, **Local Projection Consistency (LPC)** measures the smoothness and spatial coherence of the projected developmental flows in low-dimensional embeddings. Finally, **Initial/Terminal State Accuracy (ITSA)** evaluates the model’s biological relevance by quantifying its accuracy in identifying the correct developmental roots and terminally differentiated states based on the transition matrix.

### 2.2. PseudoVelo Infers Pseudo-Velocities Across Different Temporal Metrics During Erythroid Maturation

**Figure 2:**
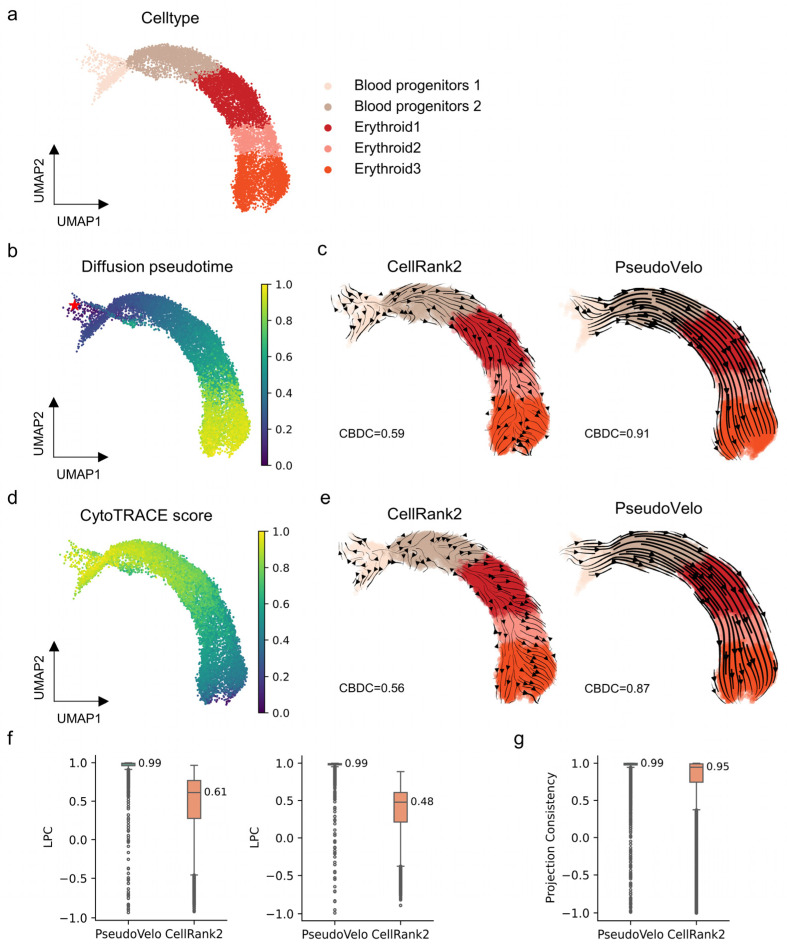
PseudoVelo infers pseudo-velocities across different temporal metrics during erythroid maturation. (**a**) UMAP embedding of gastrulation erythroid maturation (9815 cells and 2000 highly variable genes), colored by cell type. (**b**,**d**) UMAPs colored by Diffusion Pseudotime (**b**) and CytoTRACE score (**d**). The red asterisk in (**b**) indicates the root of Diffusion Pseudotime. Both metrics capture the global trajectory. (**c**,**e**), Streamline projections on the UMAP embeddings based on Diffusion Pseudotime (**c**) and CytoTRACE score (**e**). Streamlines are derived from cellular transitions inferred by CellRank 2 (**left**) and pseudo-velocities inferred by PseudoVelo (**right**). Compared to the patterns in CellRank 2, PseudoVelo consistently yields smoother and more coherent trajectories. The Cross-Boundary Direction Correctness (CBDC) metrics, calculated based on cell types to evaluate global developmental directionality, are annotated on the respective plots. (**f**) Boxplots showing the per-cell distribution of the Local Projection Consistency (LPC) metric for PseudoVelo and CellRank 2. The LPC metric measures the smoothness and spatial coherence of the projected developmental flows in low-dimensional embeddings. The left panel shows results based on Diffusion Pseudotime, and the right panel shows results based on the CytoTRACE score. (**g**) Comparison of the consistency between Diffusion Pseudotime and CytoTRACE score when used as temporal priors for inferring low-dimensional projections of developmental flows. Results derived from PseudoVelo and CellRank 2 are presented in two separate boxplots.

We applied PseudoVelo to a dataset of erythroid maturation during mouse gastrulation [15]. This developmental process serves as a classical model for evaluating cellular dynamics inference due to its continuous evolution and drastic transcriptomic remodeling. As visualized in the UMAP embedding (Figure 2a), the cells exhibit a clear linear trajectory in low-dimensional space, initiating at Blood Progenitor 1, advancing through Blood Progenitor 2 and early erythroid stages (Erythroid 1 and 2), and terminating at the Erythroid 3 state.

To drive our pseudo-velocity inference, we employed two representative inferred temporal metrics with distinct algorithmic principles: Diffusion Pseudotime (DPT) [17,18], which measures diffusion distances on a cell–cell graph, and CytoTRACE [19], which estimates developmental potential based on expressed gene counts. Although this developmental potential decreases as cells mature—meaning the CytoTRACE score is inversely correlated with the forward direction of developmental time—it can still be effectively utilized as a valid temporal proxy. Both temporal metrics successfully capture the overall global developmental trajectory (Figure 2b,d).

We then compared the performance of PseudoVelo against CellRank 2 [8] using these two distinct temporal metrics. When driven by either DPT or CytoTRACE score, CellRank 2 manages to capture the overall progression from Blood Progenitor 1 to Erythroid 3; however, the resulting trajectories are highly disordered (Figure 2c,e, left). In contrast, PseudoVelo leverages these exact same inputs to generate well-organized streamlines that exhibit robust directional consistency (Figure 2c,e, right). To quantitatively evaluate this, we computed the Cross-Boundary Direction Correctness (CBDC) metric, which assesses the accuracy of global developmental directionality between sequential cell types. PseudoVelo outperforms CellRank 2 under both temporal priors (CBDC of 0.91 vs. 0.59 for DPT and 0.87 vs. 0.56 for CytoTRACE).

Furthermore, we evaluated the Local Projection Consistency (LPC) metric, which measures the smoothness and spatial coherence of the projected developmental flows in the low-dimensional embeddings (Figure 2f). PseudoVelo consistently yields highly coherent local flows, achieving a highly median LPC of 0.99 for both DPT and CytoTRACE inputs. This surpasses CellRank 2, which exhibits much higher variance and lower median LPC scores of 0.61 and 0.48, respectively.

Finally, we assessed the robustness of the inferred developmental flows across the different temporal metrics (Figure 2g). By comparing the consistency of the projected flows when using DPT versus CytoTRACE score as temporal priors, we found that PseudoVelo maintains high stability (median consistency score of 0.99), slightly outperforming CellRank 2, which also demonstrates a relatively high consistency (median score of 0.95). Conceptually, because pseudotime metrics primarily represent the relative ordering of cells rather than real time, we adopt the designation of “pseudo-velocity.” However, the remarkable stability of these pseudo-velocities across distinct temporal inputs demonstrates that PseudoVelo captures meaningful and generalized developmental dynamics underlying these distinct pseudotimes.

### 2.3. PseudoVelo Infers Pseudo-Velocities Along Branching Trajectories in Zebrafish Embryogenesis

**Figure 3:**
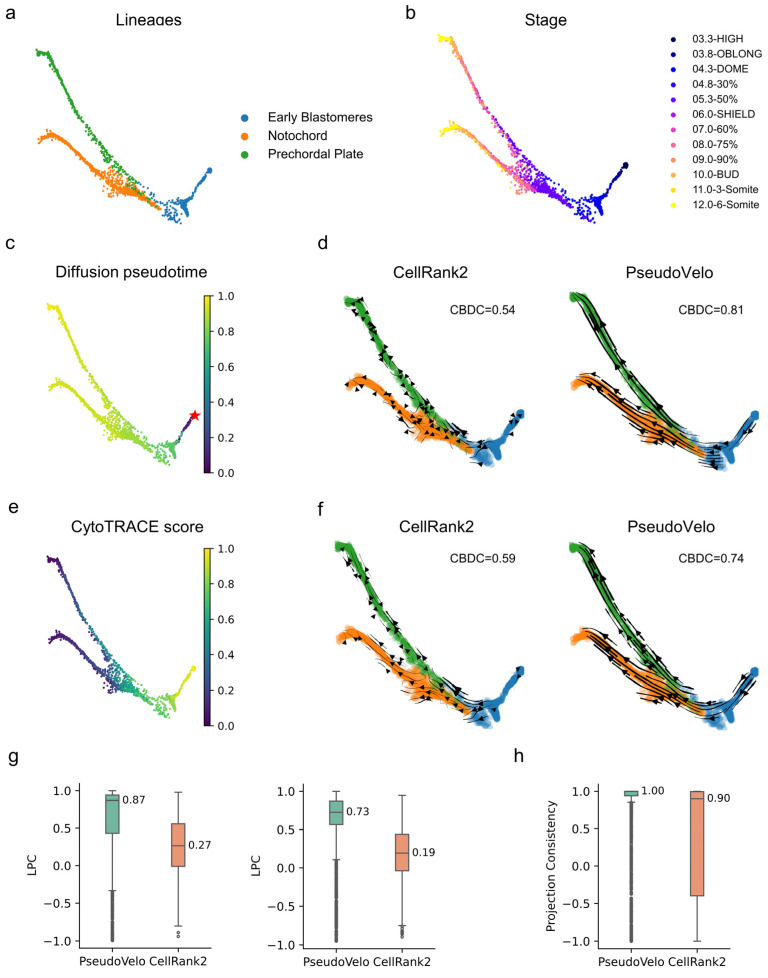
PseudoVelo infers pseudo-velocities along the branching trajectories of zebrafish embryogenesis. (**a**) Force-directed layout of the axial mesoderm lineage during zebrafish embryogenesis (2341 cells and 2000 highly variable genes), colored by cell type. (**b**) Force-directed layout colored by sampling time, serving as a reference for the trajectory direction. (**c**,**e**), Force-directed layouts colored by Diffusion Pseudotime (**c**) and CytoTRACE score (**e**). The red asterisk in (**c**) indicates the root of Diffusion Pseudotime. Both metrics capture the global trajectory. (**d**,**f**) Streamline projections on the force-directed layout based on Diffusion Pseudotime (**d**) and CytoTRACE score (**f**). Streamlines are derived from cellular transitions inferred by CellRank 2 (**left**) and pseudo-velocities inferred by PseudoVelo (**right**). Compared to the patterns in CellRank 2, PseudoVelo consistently yields smoother and more coherent trajectories. The Cross-Boundary Direction Correctness (CBDC) metrics, calculated based on sampling time to evaluate global developmental directionality, are annotated on the respective plots. (**g**) Boxplots showing the per-cell distribution of the Local Projection Consistency (LPC) metric for PseudoVelo and CellRank 2. The LPC metric measures the smoothness and spatial coherence of the projected developmental flows in low-dimensional embeddings. The left panel shows results based on Diffusion Pseudotime, and the right panel shows results based on CytoTRACE score. (**h**) Comparison of the consistency between Diffusion Pseudotime and CytoTRACE score when used as temporal priors for inferring low-dimensional projections of developmental flows. Results derived from PseudoVelo and CellRank 2 are presented in two separate boxplots.

To further evaluate PseudoVelo’s performance on more complex developmental topologies, we applied it to a branching trajectory of zebrafish early embryogenesis [14]. Specifically, we focused on the axial mesoderm lineage, a classic model for axis formation and lineage specification. In a high-resolution single-cell dataset spanning 3.3 to 12 h post-fertilization, the original force-directed layout (FDL) clearly captures the bifurcation of early blastomeres into two distinct terminal fates: the notochord and the prechordal plate (Figure 3a). This branching progression is further validated by the continuous experimental time points (Figure 3b).

As in the previous analysis, we drove the pseudo-velocity inference using two distinct temporal metrics: Diffusion Pseudotime (DPT) [17,18] and CytoTRACE score [19] (Figure 3c,e). We observed that the performance of CellRank 2 [8] is highly sensitive to the choice of input metric on this branching manifold. When driven by DPT—which exhibits a skewed temporal distribution in this specific dataset—CellRank 2 produces highly disordered and chaotic trajectories that fail to clearly capture the developmental progression (Figure 3d, left). Although CellRank 2 performs noticeably better with the CytoTRACE score by capturing the global branching topology, the resulting streamlines remain somewhat disordered (Figure 3f, left). In contrast, PseudoVelo effectively overcomes the skewed distribution of DPT and generates smooth, coherent trajectories with both temporal inputs, accurately reconstructing the developmental bifurcation toward both the notochord and prechordal plate (Figure 3d,f, right). To quantitatively evaluate this, we computed the Cross-Boundary Direction Correctness (CBDC) metric based on the experimental sampling time. PseudoVelo consistently outperforms CellRank 2 under both temporal priors, achieving CBDC of 0.81 versus 0.54 for DPT, and 0.74 versus 0.59 for CytoTRACE score.

Furthermore, we evaluated the Local Projection Consistency (LPC) metric to measure the smoothness and spatial coherence of the projected developmental flows (Figure 3g). PseudoVelo successfully maintains coherent local flows even on this complex branching manifold, achieving median LPC scores of 0.87 and 0.73 for DPT and CytoTRACE inputs, respectively. In contrast, CellRank 2 exhibits much lower median LPC scores of 0.27 and 0.19.

Finally, we assessed the robustness of the inferred developmental flows across the different temporal metrics (Figure 3h). By comparing the consistency of the projected flows when using DPT versus CytoTRACE score as temporal priors, PseudoVelo demonstrates high stability with a median consistency score of 1.00. CellRank 2 also displays a relatively high consistency across temporal inputs (median score of 0.90). The success of PseudoVelo on this branching manifold highlights the efficacy of its adaptive fitting and numerical differentiation approach.

### 2.4. PseudoVelo Demonstrates Robustness Across Various Data and Perturbations

To further benchmark the performance of PseudoVelo on complex datasets, we expanded our evaluation to two additional classical datasets: a mouse pancreatic endocrinogenesis dataset (referred to as Pancreas in the tables) [16]; and a dataset of early human hematopoiesis from CD34+ bone marrow cells (referred to as Bone Marrow) [9] (Table 1, Figures S1 and S2). When provided with a correct temporal prior, PseudoVelo consistently outperformed CellRank 2 across these datasets in terms of Cross-Boundary Direction Correctness (CBDC) and Local Projection Consistency (LPC). Initial/Terminal State Accuracy (ITSA), which was calculated using the CellRank framework based on the inferred transition matrices, also confirmed that PseudoVelo effectively identifies the biological roots and terminal states. Notably, to calculate the CBDC metric, we utilized sequential cell types for the Pancreas, Bone Marrow, and Erythroid datasets, whereas we used the experimental sampling time for the Zebrafish embryogenesis dataset.

To systematically assess model robustness, we evaluated both methods under three data perturbation strategies: re-clustering (using Scanpy’s Leiden algorithm [18,20] for unsupervised clustering, which PseudoVelo leverages for sub-population adaptive fitting), cell subsampling (randomly retaining 50% of cells), and gene subsampling (randomly retaining 50% of genes) (Table 2, Figure S3). The re-clustering results demonstrate that PseudoVelo does not rely on predefined or specific manual annotations; performing unsupervised clustering still allows it to run effectively, with the final performance remaining almost unaffected. For the Erythroid maturation dataset (previously analyzed in Section 2.2), which presents a linear non-branching trajectory, PseudoVelo does not rely on cluster information, and thus its re-clustering results naturally remained perfectly consistent with the original unperturbed baseline. Across all datasets, including the Zebrafish embryogenesis (Section 2.3), Pancreas, and Bone Marrow datasets, PseudoVelo demonstrated high resilience to these perturbations, maintaining superior CBDC and LPC scores compared to CellRank 2.

In the Pancreas and Bone Marrow datasets, the CytoTRACE score provides an incorrect representation of the developmental direction. As shown in Table 1, PseudoVelo reflects this erroneous input with a substantial drop in performance metrics, directly illustrating the method’s effective mapping of the provided temporal prior. In contrast, CellRank 2 yields CBDC scores hovering around 0.5 with only a slight decrease, indicative of severe random noise. We further validated this responsiveness by calculating DPT using sub-optimal and incorrect root nodes (root2 and root3) in the Zebrafish embryogenesis dataset (Table S1, Figure S4). Similarly to the CytoTRACE results, supplying these incorrect root nodes caused a prominent and expected decline in PseudoVelo’s CBDC, whereas CellRank 2 showed only a marginal decrease.

Finally, while traditional RNA velocity methods are based on fundamentally different principles (utilizing spliced/unspliced ratios rather than pseudotime mapping) and are not our direct comparison targets, we computed the metrics for scVelo [2] and Cell2fate [5] on the Erythroid maturation and Pancreas datasets to provide a baseline reference (Table S2, Figure S5). Notably, PseudoVelo generates highly coherent flow fields with LPC scores highly comparable to these traditional RNA velocity methods. In contrast, CellRank 2 produces much lower LPC scores, further highlighting PseudoVelo’s advantage in producing smooth developmental flows in the form of pseudo-velocities. Additionally, we evaluated the computational efficiency of our method, detailing the runtime of PseudoVelo in Table S3.

## 3. Discussion

The inference of cellular dynamics from single-cell RNA sequencing data remains a fundamental challenge in developmental biology. While RNA velocity [1,2] has provided a valuable framework for predicting future cell states, its reliance on splicing kinetics can sometimes lead to conflicting trajectories, particularly in complex systems. Pseudotime-based alternative approaches, such as CellRank 2 [8], have offered useful workarounds but estimate transitions by biasing nearest-neighbor graphs rather than deriving transcriptomic rates of change. In this study, we introduced PseudoVelo, a computational framework that infers pseudo-velocity from pseudotime. Building upon the conventional pseudotime fitting of gene expression dynamics based on Generalized Additive Models (GAMs), PseudoVelo employs central difference approximation to estimate the rate of change for each gene, yielding a pseudo-velocity matrix. Importantly, this matrix is fully compatible with existing downstream analysis tools originally designed for traditional RNA velocity, ensuring seamless integration into standard single-cell workflows.

Our evaluations across multiple datasets, including erythrocyte maturation, zebrafish embryogenesis, mouse pancreatic endocrinogenesis, and human hematopoiesis, highlight the utility of PseudoVelo. Quantitative benchmarking using metrics such as Cross-Boundary Direction Correctness (CBDC) and Local Projection Consistency (LPC) demonstrated that, compared to the graph-biasing approach of CellRank 2 [8], PseudoVelo generally produced more organized developmental flows with stronger local spatial coherence, mitigating the disordered transitions sometimes observed in graph-based estimations. Additionally, our perturbation analyses (re-clustering, cell subsampling, and gene subsampling) indicate that PseudoVelo maintains a stable performance profile across various data alterations.

We also acknowledge several potential concerns regarding PseudoVelo. First, because the model is fundamentally driven by pseudotime, there are natural apprehensions regarding its dependence on the scale and quality of the initial temporal prior. Regarding the temporal scale, our evaluations demonstrate that PseudoVelo effectively overcomes this challenge; by utilizing distinct temporal priors with varying temporal distributions (such as DPT [17] and CytoTRACE [19]), the model yields highly consistent performance. Conversely, the quality of the prior remains a strict limitation. As demonstrated in our results, if an incorrect temporal prior is supplied, PseudoVelo cannot correct this upstream failure and will predictably reflect the error. Second, regarding the subpopulation-aware adaptive fitting mechanism, one might be concerned that it relies heavily on specific, user-provided lineage annotations. However, our re-clustering perturbation tests alleviate this concern, demonstrating that the framework does not strictly depend on predefined manual annotations; employing standard unsupervised clustering algorithms (such as Leiden [20]) serves as a highly adequate substitute, yielding performance that is equally effective. Finally, while GAMs are highly flexible, modeling massive datasets with tens of thousands of genes across complex manifolds can be computationally intensive. To address this, our implementation utilizes multi-process parallel optimization to significantly mitigate the computational burden. Furthermore, we have provided an empirical evaluation of PseudoVelo’s runtime in the Supplementary Materials.

## 4. Methods

### 4.1. PseudoVelo Algorithm Design

PseudoVelo is a computational framework designed to infer pseudo-velocity driven by pseudotime. The core principle of the algorithm is to construct a continuous statistical mapping between pseudotime and gene expression and subsequently utilize numerical differentiation to estimate the pseudo-velocity.

### 4.2. Statistical Modeling of Expression Dynamics

The core objective of our algorithm is to map high-dimensional, discrete single-cell expression counts into a continuous probability space driven by a pseudotime covariate. To address the overdispersion commonly observed in single-cell RNA sequencing data, we model the conditional distribution of gene expression using the Negative Binomial (NB) distribution [21].

For a given single-cell dataset, let n be the total number of cells and let m be the total number of genes. Let $Y_{ig}$ represent the transcript abundance of gene g (g=1,…,m) in cell i (i=1,…,n). Concurrently, each cell is assigned a pseudotime coordinate $t_{i}\in R$. Within our Generalized Additive Model (GAM) framework, the conditional distribution of gene expression is parameterized as

$$Y_{ig}|t_{i}∼NB(\mu_{ig}\left(t_{i}\right),\sigma_{ig}(t_{i}))$$

where the mean parameter $\mu_{ig}$ represents the basal expression level of gene g at time $t_{i}$ and the dispersion parameter $\sigma_{ig}$ accounts for both biological and technical stochasticity.

To preserve biological non-linear dynamics while ensuring the continuous differentiability of the function, we employ a log link function to associate the mean parameter $\mu_{ig}$ with the pseudotime $t_{i}$:

$$log(\mu_{ig}(t_{i}))=\beta_{g0}+f_{g}(t_{i})$$

Here, $\beta_{g0}$ serves as the intercept for the baseline expression level, and the smooth function $f_{g}(t_{i})$ is non-linearly approximated through an expansion of B-spline basis functions:

$$f_{g}(t_{i})=\sum_{k=1}^{K_{g}}b_{gk}(t_{i})\gamma_{gk}$$

where $b_{gk}(\cdot )$ represents the spline basis function of degree d (defaulted to d=3). The parameter $K_{g}$ represents the degrees of freedom, which dictate the flexibility of the curve. Unlike traditional GAMs that use a fixed global degree of freedom for all cells, PseudoVelo introduces a dynamic knot allocation mechanism tailored for cellular sub-clusters (detailed in Section 4.4).

**Model Implementation:**

The GAM framework was implemented using the statsmodels package (v[0.14.5]) in Python, specifically utilizing the GLMGam and BSplines modules. Our primary objective is to obtain a point estimate of the mean expression trajectory μ(t) to derive velocity. Statistically, the point estimates of the mean are highly robust and remain consistent regardless of the exact dispersion value. Therefore, to ensure algorithmic stability and optimize computational runtime, we utilized a fixed dispersion parameter (α=1) as a baseline overdispersion penalty. This approach successfully regularizes the non-linear mean fitting without sacrificing computational efficiency.

PseudoVelo is designed to be highly scalable. Since the GAM fitting processes are strictly independent across genes, multi-core parallelization was implemented via the joblib package (v[1.5.1]) [22]. This parallel processing significantly reduces the runtime overhead.

### 4.3. Velocity Derivation via Numerical Differentiation

Once a continuous function $\mu_{g}(t)$ describing the mean expression of each gene with respect to pseudotime is obtained, pseudo-velocity can be defined as the instantaneous rate of change in the expected expression value over time.

The theoretical solution for pseudo-velocity is

$$v_{ig}={\left.\frac{d\mu_{g}(t)}{dt}\right|}_{t=t_{i}}$$

To approximate this derivative in a discrete computational environment and avoid numerical instability from direct differentiation, we employ a central difference approximation scheme. For a specific, infinitesimally small time perturbation δ, the velocity component is approximately calculated as

$$v_{ig}\approx \frac{\mu_{g}(t_{i}+\delta )-\mu_{g}(t_{i}-\delta )}{2\delta }$$

In our implementation, the perturbation δ is not arbitrarily assigned but is strictly tied to the global time span of the entire manifold. Letting the upper and lower limits of the global time interval be $t_{max}$ and $t_{min}$ respectively, we define $\delta =\epsilon \cdot (t_{max}-t_{min})$, where the scaling factor ϵ defaults to $10^{-6}$. This adaptive perturbation ensures the validity of the local limit theorem for numerical differentiation while preventing computational overflow or underflow caused by floating-point precision limitations across different time scales.

**Boundary Handling:**

To strictly prevent the perturbation bounds from exceeding the empirical pseudotime domain, a boundary clipping strategy was implemented. Specifically, the perturbed coordinates $t_{i}-\delta$ and $t_{i}+\delta$ are constrained using a min--max truncation, ensuring that any boundary predictions are conservatively evaluated at the absolute minimum or maximum of the observed pseudotime within the local cluster.

### 4.4. Adaptive Fitting Mechanism for Cell Subpopulations

In real-world developmental systems, a single global time axis often fails to capture the heterogeneous dynamic features across different cell lineages. Therefore, PseudoVelo integrates an adaptive fitting mechanism tailored for cell subpopulations.

When single-cell data is accompanied by lineage or cluster labels, the algorithm decomposes the probability mapping—originally performed on the global domain—into multiple independent subspace fitting tasks. For a set of cells $C_{c}$ belonging to a specific cluster c, the internal time span is typically only a subset of the global span. To accommodate this, we defined a global baseline degree of freedom ($K_{base}$, set to 10 by default). The algorithm adaptively adjusts the flexibility parameter of the spline function:

$$K_{g}^{\left(c\right)}=max\left(\left\lceil K_{base}\cdot \frac{\Delta t_{c}}{\Delta t_{global}}\right\rceil ,d+1\right)$$

where $\Delta t_{c}$ is the effective time span of the subset and $\Delta t_{global}$ is the global time span. This constraint mathematically ensures that the degrees of freedom always satisfy the statistical lower bound ($K_{g}\geq d+1$). More importantly, this dynamic scaling strategy ensures that in terminally differentiated clusters with short time spans, the model favors more robust, lower-order smoothing to prevent biologically implausible oscillations. Conversely, in the main developmental trunk where the time span is longer and transcription changes drastically, the model is granted sufficient degrees of freedom to capture complex dynamics.

Furthermore, for sub-clusters that are completely quiescent or whose time span approaches zero, the algorithm triggers a safeguard mechanism, directly downgrading them to a constant mapping (constant expression and zero velocity), thereby ensuring the robustness of the entire computational pipeline.

### 4.5. scRNA-Seq Data and Pre-Processing

Single-cell RNA-seq data were pre-processed prior to GAM fitting using the Scanpy package (v[1.11.4]) [18]. The input expression matrix (X) was library-size-normalized and log-transformed. Subsequently, to ensure statistical reliability and reduce computational redundancy, highly variable genes (HVGs) were calculated, and the top 2000 HVGs were selected for the downstream velocity modeling pipeline.

We analyzed four datasets: a zebrafish embryogenesis subset of 2341 cells (Farrell et al. [14], filtered according to Lange et al. [23]), a mouse gastrulation erythroid lineage subset of 9815 cells provided by the scVelo documentation [2] (originally from Pijuan-Sala et al. [15]), a human bone marrow CD34+ dataset provided by CellRank [8] (originally from Setty et al. [9]), and a mouse pancreatic endocrinogenesis dataset provided by scVelo [2] (originally from Bastidas-Ponce et al. [16]). The complete code for data pre-processing and reproducibility can be found via the GitHub link in the Data and Code Availability section.

### 4.6. Embedding Projection and Visualization

Once the high-dimensional velocities were estimated via our framework, downstream graph construction and embedding projections were performed utilizing the scvelo package (v[0.3.3]) [2]. Specifically, a transition probability graph was constructed by computing cosine similarities between cell-to-cell transitions and the inferred velocity vectors using scvelo.tl.velocity_graph. The transition probability graph was then projected onto low-dimensional embeddings (e.g., UMAP) as stream plots using scvelo.pl.velocity_embedding_stream.

### 4.7. Benchmarking Metrics

To quantitatively assess the performance and reliability of PseudoVelo and other comparative methods, we designed and employed three evaluation metrics: Cross-Boundary Direction Correctness (CBDC), Local Projection Consistency (LPC), and Initial/Terminal State Accuracy (ITSA). It is important to note that PseudoVelo outputs predicted pseudo-velocities, whereas our primary comparative method, CellRank 2, directly outputs a cell-to-cell transition probability matrix. Because standard downstream analyses of velocities require their conversion into a cell-to-cell transition probability matrix, all our proposed metrics are evaluated at the transition probability matrix level. Specifically, for PseudoVelo and other velocity-based methods, we input the predicted (pseudo-)velocities into scVelo to compute the cell-to-cell transition probability matrix T. For CellRank 2, we directly utilize its output cell-to-cell transition probability matrix T.

**Cross-Boundary Direction Correctness (CBDC):**

The CBDC metric is designed to evaluate whether the inferred developmental flows accurately capture the expected directionality between sequential cell clusters along a known biological lineage. Given a predefined ground-truth lineage composed of ordered cell clusters $\left(C_{1}\to C_{2}\to \cdots \to C_{k}\right)$, we define correct (forward) transition edges as $E_{correct}=\{(C_{i},C_{i+1})\}$ and incorrect (backward) transition edges as $E_{wrong}=\{(C_{i+1},C_{i})\}$.

Using the transition probability matrix T, the global weight of correct transitions ($W_{correct}$) and wrong transitions ($W_{wrong}$) are calculated by summing the transition probabilities $T_{uv}$ for all cell pairs (u,v) where cell u and cell v belong to the clusters associated with $E_{correct}$ and $E_{wrong}$, respectively. The CBDC score is then defined as

$$CBDC=\frac{W_{correct}}{W_{correct}+W_{wrong}}$$

A higher CBDC score (approaching 1.0) indicates that the cell state transitions across cluster boundaries are highly concordant with the true biological developmental direction.

**Local Projection Consistency (LPC) and Cross-Temporal Projection Consistency:**

To evaluate the smoothness and spatial coherence of the projected developmental flows in a low-dimensional embedding (e.g., UMAP), we introduced the Local Projection Consistency (LPC) metric. First, utilizing the transition probability matrix T, we calculate the expected velocity vector $v_{i}$ for each cell i in the low-dimensional embedding space.

Subsequently, relying on the structural k-nearest neighbor (k-NN) connectivity graph—which is computed via Scanpy based on the transcriptomic similarities among cells—we calculate the cosine similarity between the expected velocity of a reference cell and those of its topological neighbors. For a given cell i with its neighborhood set N(i) defined by the connectivity graph, its local consistency score is

$$LPC_{i}=\frac{1}{\left|N(i)\right|}\sum_{j\in N(i)}cos(v_{i},v_{j})$$

The global LPC score is obtained by calculating the median of $LPC_{i}\;$ across all valid cells. A high LPC value suggests that neighboring cells share similar developmental vector orientations, reflecting a robust and locally stable vector field projection without chaotic vectors.

Building upon this framework, to align projected flows across different temporal inputs, we introduced a variant metric: **Cross-Temporal Projection Consistency**. For a given cell i, we compute its expected velocity vectors, $v_{1,i}$ and $v_{2,i}$, utilizing the distinct transition probability matrices from the two temporal inputs, respectively. The metric is directly defined as the cosine similarity between these two condition-specific vectors:

$${\mathrm{Cross}-\mathrm{Temporal}\;\mathrm{Projection}\;\mathrm{Consistency}}_{i}=cos(v_{1,i},v_{2,i})$$

A high value in this metric indicates that the inferred developmental trajectory for an individual cell remains robust regardless of the varying temporal inputs.

**Initial/Terminal State Accuracy (ITSA):**

To assess the biological relevance of the inferred macroscopic trajectory, the ITSA metric evaluates the capacity to correctly identify developmental roots (initial states) and terminally differentiated populations (terminal states).

We feed the transition probability matrix T into CellRank to computationally infer the macrostates (initial and terminal states). Using the biological ground truth as a reference, the accuracy is calculated as the ratio of correctly identified state cells (the intersection of predicted and true cells) to the size of the union of predicted and true state cell sets. Mathematically, it operates similarly to a Jaccard Index:

$$ITSA=\frac{\left|S_{pred}\cap S_{true}\right|}{\left|S_{pred}\cup S_{true}\right|}$$

where $S_{pred}$ represents the subset of cells predicted as initial/terminal states and $S_{true}$ represents the true biological initial/terminal subsets. This metric robustly penalizes both false positives and false negatives, ensuring that the inferred macroscopic manifold starts and ends at biologically correct cell subpopulations.

## 5. Conclusions

PseudoVelo provides a straightforward solution for inferring cellular dynamics, representing a novel computational tool for developmental biology research. By moving beyond simplified splicing kinetics and graph manipulations, PseudoVelo equips researchers with an effective framework to unravel the complex transcriptional dynamics underlying cellular differentiation, embryogenesis, and broader developmental processes.

## Figures and Tables

**Table 1:** Quantitative benchmarking of PseudoVelo and CellRank 2 across multiple developmental datasets.

Dataset	Pseudotime Prior	Prior Status	PseudoVelo			CellRank2		
			CBDC	LPC (Median)	ITSA	CBDC	LPC (Median)	ITSA
Erythroid maturation	DPT	Correct	**0.91**	**0.99**	**1**	0.59	0.61	0.67
Erythroid maturation	CytoTRACE	Correct	**0.87**	**0.99**	**1**	0.56	0.48	0.67
Zebrafish embryogenesis	DPT	Correct	**0.81**	**0.87**	**0.75**	0.54	0.27	**0.75**
Zebrafish embryogenesis	CytoTRACE	Correct	**0.74**	**0.73**	**1**	0.59	0.19	0.75
Pancreas	DPT	Correct	**0.63**	**0.82**	**0.8**	0.53	0.52	0.57
Pancreas	CytoTRACE	Incorrect	0.4	**0.72**	0.14	**0.5**	0.51	**0.57**
Bone Marrow	DPT	Correct	**0.69**	**0.46**	**1**	0.57	0.29	0.63
Bone Marrow	CytoTRACE	Incorrect	**0.55**	**0.53**	0.33	0.52	0.35	**0.63**

Performance is evaluated using three metrics: Cross-Boundary Direction Correctness (CBDC) for global developmental directionality, Local Projection Consistency (LPC, median) for the smoothness of the projected developmental flows, and Initial/Terminal State Accuracy (ITSA) for the biological relevance of cell fate prediction. Two temporal priors, Diffusion Pseudotime (DPT) and CytoTRACE score, were utilized. Yellow shading indicates an incorrect input temporal prior. Bold values indicate the superior performance between the two methods for a given metric and condition.

**Table 2:** Robustness benchmarking of PseudoVelo and CellRank 2 under various data perturbations.

Dataset	Pseudotime Prior	Perturbation	PseudoVelo			CellRank2		
			CBDC	LPC (Median)	ITSA	CBDC	LPC (Median)	ITSA
Erythroid maturation	DPT	Re-clustering	**0.91**	**0.99**	**1**	0.59	0.61	0.67
Erythroid maturation	DPT	Cell Subsampling	**0.93**	**0.99**	**1**	0.6	0.66	0.67
Erythroid maturation	DPT	Gene Subsampling	**0.89**	**0.99**	**1**	0.59	0.6	0.67
Zebrafish embryogenesis	DPT	Re-clustering	**0.77**	**0.72**	**0.75**	0.54	0.27	**0.75**
Zebrafish embryogenesis	DPT	Cell Subsampling	**0.8**	**0.79**	**0.75**	0.54	0.36	**0.75**
Zebrafish embryogenesis	DPT	Gene Subsampling	**0.78**	**0.89**	**0.75**	0.53	0.25	**0.75**
Pancreas	DPT	Re-clustering	**0.65**	**0.72**	**1**	0.53	0.52	0.57
Pancreas	DPT	Cell Subsampling	**0.61**	**0.68**	**0.5**	0.54	0.63	0.38
Pancreas	DPT	Gene Subsampling	**0.61**	**0.75**	**0.8**	0.53	0.46	0.57
Bone Marrow	DPT	Re-clustering	**0.72**	**0.65**	**0.71**	0.57	0.29	0.63
Bone Marrow	DPT	Cell Subsampling	**0.68**	0.31	**0.71**	0.57	**0.38**	0.63
Bone Marrow	DPT	Gene Subsampling	**0.72**	**0.61**	**0.83**	0.59	0.36	0.44

To assess the stability of model performance, both methods were evaluated under three data perturbation strategies: re-clustering, cell subsampling, and gene subsampling. Diffusion Pseudotime (DPT) was utilized as the consistent temporal prior across all four datasets. Model robustness is quantitatively evaluated using three metrics: Cross-Boundary Direction Correctness (CBDC) for global developmental directionality, Local Projection Consistency (LPC, median) for the smoothness of the projected developmental flows, and Initial/Terminal State Accuracy (ITSA) for the biological relevance of cell fate prediction. Bold values indicate the superior performance between the two methods for a given metric and perturbation condition.

## Data Availability

The zebrafish embryogenesis data used in this study can be accessed via CellRank (cellrank.datasets.zebrafish()), with the raw data available at GEO: GSE106587. The erythrocyte maturation data can be obtained via scVelo (scvelo.datasets.gastrulation_erythroid()), with the raw data available at ArrayExpress: E-MTAB-6967. The human bone marrow data can be accessed via CellRank (cellrank.datasets.bone_marrow()), with the raw data available at BioStudies: S-SUBS8. The mouse pancreatic endocrinogenesis data can be obtained via scVelo (scvelo.datasets.pancreas()), with the raw data available at GEO: GSE132188. PseudoVelo is released under the BSD-3-Clause license. The source code, data, and reproducibility scripts can be found at https://github.com/hsinring/pseudovelo (accessed on 16 July 2026).

## Supplementary Materials

The following supporting information can be downloaded at: https://www.mdpi.com/article/10.3390/ijms27146420/s1.

## References

[1] La Manno G., Soldatov R., Zeisel A., Braun E., Hochgerner H., Petukhov V., Lidschreiber K., Kastriti M.E., Lönnerberg P., Furlan A. (2018). RNA velocity of single cells. Nature.

[2] Bergen V., Lange M., Peidli S., Wolf F.A., Theis F.J. (2020). Generalizing RNA velocity to transient cell states through dynamical modeling. Nat. Biotechnol..

[3] Li S., Zhang P., Chen W., Ye L., Brannan K.W., Le N.-T., Abe J.-I., Cooke J.P., Wang G. (2024). A relay velocity model infers cell-dependent RNA velocity. Nat. Biotechnol..

[4] Gayoso A., Weiler P., Lotfollahi M., Klein D., Hong J., Streets A., Theis F.J., Yosef N. (2024). Deep generative modeling of transcriptional dynamics for RNA velocity analysis in single cells. Nat. Methods.

[5] Aivazidis A., Memi F., Kleshchevnikov V., Er S., Clarke B., Stegle O., Bayraktar O.A. (2025). Cell2fate infers RNA velocity modules to improve cell fate prediction. Nat. Methods.

[6] Bergen V., Soldatov R.A., Kharchenko P.V., Theis F.J. (2021). RNA velocity—Current challenges and future perspectives. Mol. Syst. Biol..

[7] Gorin G., Fang M., Chari T., Pachter L. (2022). RNA velocity unraveled. PLoS Comput. Biol..

[8] Weiler P., Lange M., Klein M., Pe’er D., Theis F. (2024). CellRank 2: Unified fate mapping in multiview single-cell data. Nat. Methods.

[9] Setty M., Kiseliovas V., Levine J., Gayoso A., Mazutis L., Pe’er D. (2019). Characterization of cell fate probabilities in single-cell data with Palantir. Nat. Biotechnol..

[10] Wood S.N. (2017). Generalized Additive Models: An Introduction with R.

[11] Qiu X., Mao Q., Tang Y., Wang L., Chawla R., Pliner H.A., Trapnell C. (2017). Reversed graph embedding resolves complex single-cell trajectories. Nat. Methods.

[12] Van den Berge K., de Bézieux H.R., Street K., Saelens W., Cannoodt R., Saeys Y., Dudoit S., Clement L. (2020). Trajectory-based differential expression analysis for single-cell sequencing data. Nat. Commun..

[13] Song D., Wang Q., Yan G., Liu T., Sun T., Li J.J. (2024). scDesign3 generates realistic in silico data for multimodal single-cell and spatial omics. Nat. Biotechnol..

[14] Farrell J.A., Wang Y., Riesenfeld S.J., Shekhar K., Regev A., Schier A.F. (2018). Single-cell reconstruction of developmental trajectories during zebrafish embryogenesis. Science.

[15] Pijuan-Sala B., Griffiths J.A., Guibentif C., Hiscock T.W., Jawaid W., Calero-Nieto F.J., Mulas C., Ibarra-Soria X., Tyser R.C.V., Ho D.L.L. (2019). A single-cell molecular map of mouse gastrulation and early organogenesis. Nature.

[16] Bastidas-Ponce A., Tritschler S., Dony L., Scheibner K., Tarquis-Medina M., Salinno C., Schirge S., Burtscher I., Böttcher A., Theis F.J. (2019). Comprehensive single cell mRNA profiling reveals a detailed roadmap for pancreatic endocrinogenesis. Development.

[17] Haghverdi L., Büttner M., Wolf F.A., Buettner F., Theis F.J. (2016). Diffusion pseudotime robustly reconstructs lineage branching. Nat. Methods.

[18] Wolf F.A., Angerer P., Theis F.J. (2018). SCANPY: Large-scale single-cell gene expression data analysis. Genome Biol..

[19] Gulati G.S., Sikandar S.S., Wesche D.J., Manjunath A., Bharadwaj A., Berger M.J., Ilagan F., Kuo A.H., Hsieh R.W., Cai S. (2020). Single-cell transcriptional diversity is a hallmark of developmental potential. Science.

[20] Traag V.A., Waltman L., van Eck N.J. (2019). From Louvain to Leiden: Guaranteeing well-connected communities. Sci. Rep..

[21] Svensson V. (2020). Droplet scRNA-seq is not zero-inflated. Nat. Biotechnol..

[22] Joblib Developers Joblib: Running Python Functions as Pipeline Jobs. https://joblib.readthedocs.io/.

[23] Lange M., Bergen V., Klein M., Setty M., Reuter B., Bakhti M., Lickert H., Ansari M., Schniering J., Schiller H.B. (2022). CellRank for directed single-cell fate mapping. Nat. Methods.

