# Supplementary material for: PseudoVelo: Inferring Gene Expression Derivatives Along Pseudotime as Pseudo-Velocity
Source: Int J Mol Sci. 2026 Jul 19;27(14):6420. doi: 10.3390/ijms27146420 (PMC13410147; doi:10.3390/ijms27146420)
Supplement: Supplementary file 1 [file ijms-27-06420-s001.zip › ijms-4423845-supplementary.pdf]

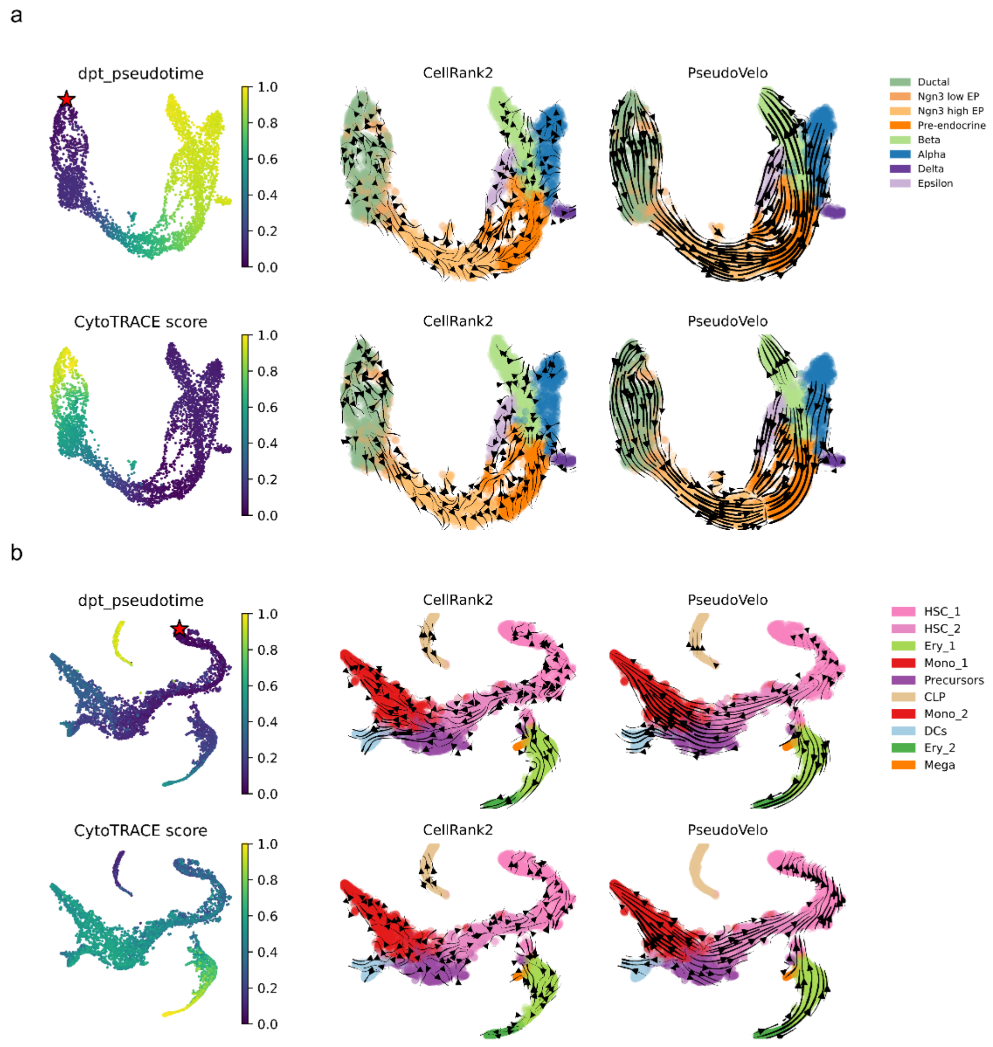

Figure S1. PseudoVelo infers pseudo-velocities across different temporal metrics in additional developmental datasets.

a, Application to the mouse pancreatic endocrinogenesis dataset (3,686 cells and 2,000 highly variable genes) visualized on UMAP embeddings. The top row shows the UMAP colored by Diffusion Pseudotime (left), alongside streamline projections on the UMAP embeddings based on Diffusion Pseudotime derived from cellular transitions inferred by CellRank 2 (middle) and pseudo-velocities inferred by PseudoVelo (right). The bottom row shows the UMAP colored by CytoTRACE score (left), alongside the corresponding streamline projections based on CytoTRACE score derived from CellRank 2 (middle) and PseudoVelo (right). b, Application to the early human hematopoiesis dataset (5,780 cells and 2,000 highly variable genes) visualized on t-SNE embeddings. Similar to (a), the top row displays the t-SNE colored by Diffusion Pseudotime (left) and the subsequent streamline projections inferred by CellRank 2 (middle) and PseudoVelo (right). The bottom row displays the t-SNE colored by CytoTRACE score (left) and the corresponding streamline projections by CellRank 2 (middle) and PseudoVelo (right). For all relevant panels in (a) and (b), the red asterisk indicates the root of Diffusion Pseudotime. Notably, only Diffusion Pseudotime successfully captures the correct global developmental trajectory in these datasets, whereas the CytoTRACE score yields an incorrect temporal direction.

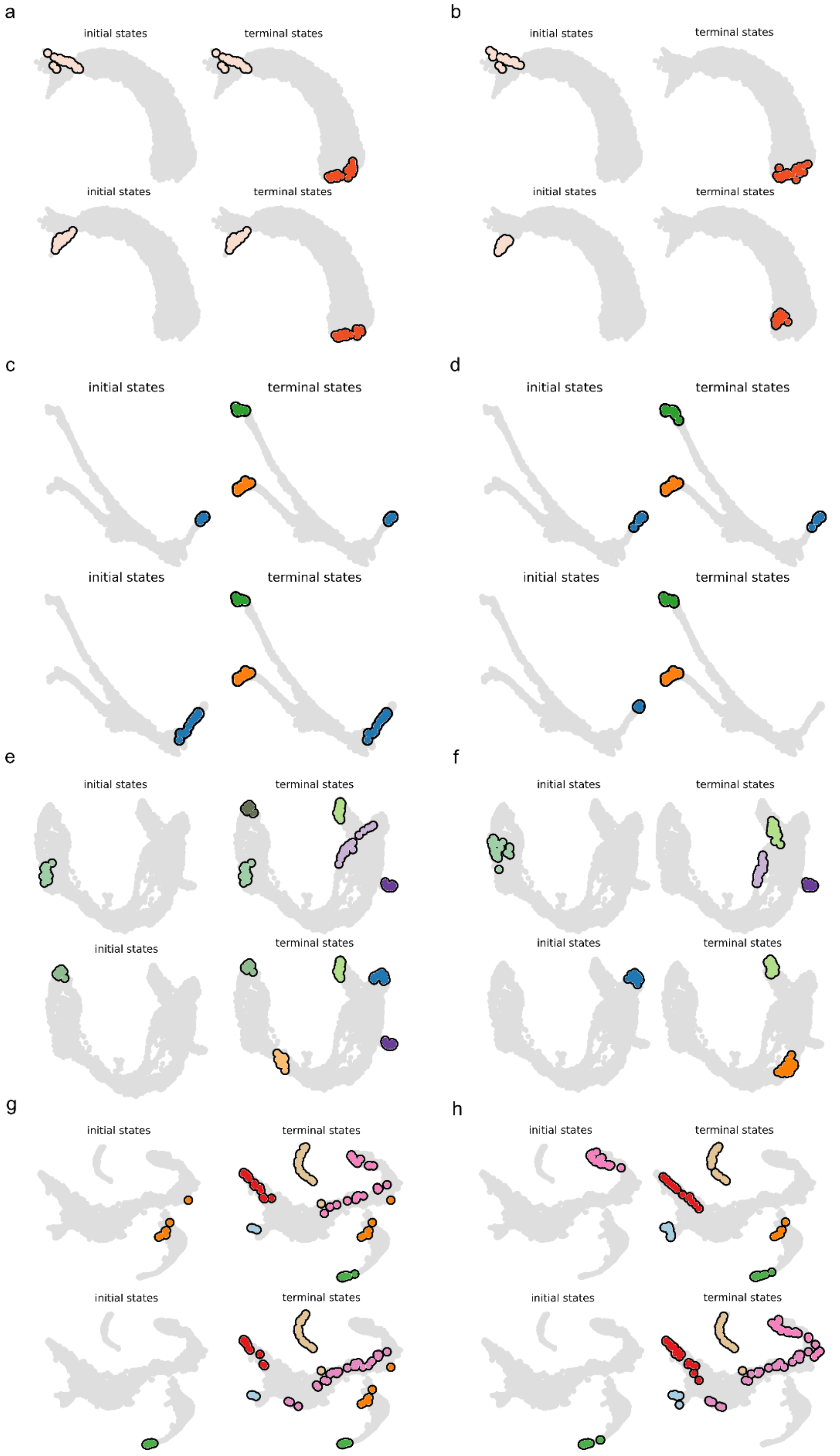

Figure S2. Identification of initial and terminal states based on transition probabilities inferred by PseudoVelo and CellRank 2 across multiple developmental datasets.

Initial and terminal states were identified using CellRank based on the cell-to-cell transition

probability matrices predicted by CellRank 2 and PseudoVelo. These visualizations correspond to the Initial/Terminal State Accuracy (ITSA) evaluation presented in Table 1. a, b, Erythroid maturation dataset. State identification based on the transition probabilities derived from CellRank 2 (a) and PseudoVelo (b). c, d, Zebrafish embryogenesis dataset. State identification based on the transition probabilities derived from CellRank 2 (c) and PseudoVelo (d). e, f, Mouse pancreatic endocrinogenesis dataset. State identification based on the transition probabilities derived from CellRank 2 (e) and PseudoVelo (f). g, h, Early human hematopoiesis (bone marrow) dataset. State identification based on the transition probabilities derived from CellRank 2 (g) and PseudoVelo (h). For each panel (a–h), there are four sub-panels arranged in two rows and two columns: the top row utilizes Diffusion Pseudotime as the temporal prior, while the bottom row utilizes the CytoTRACE score. Within each row, the left sub-panel displays the identified initial states, and the right sub-panel displays the identified terminal states.

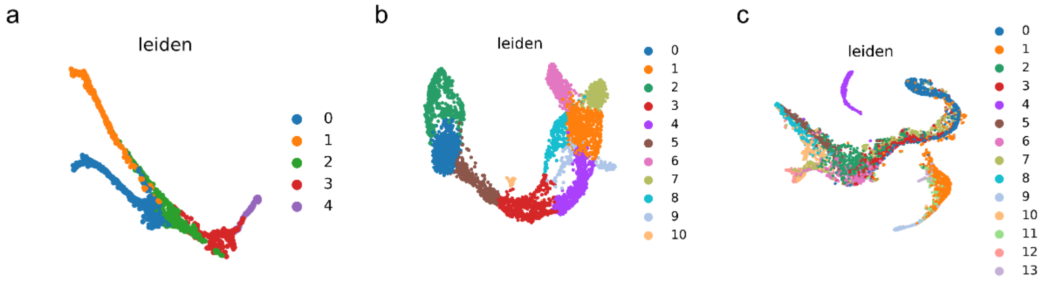

Figure S3. Unsupervised re-clustering results of developmental datasets for robustness benchmarking.

These visualizations correspond to the "Re-clustering" data perturbation strategy evaluated in Table 2. Unsupervised re-clustering was performed using the Leiden algorithm implemented in Scanpy. The resolution parameter for each dataset was manually adjusted to ensure that the number of identified clusters falls within a biologically reasonable range. a, Re-clustering results for the zebrafish embryogenesis dataset, using a Leiden resolution of 0.2. b, Re-clustering results for the mouse pancreatic endocrinogenesis dataset, using a Leiden resolution of 0.8. c, Re-clustering results for the early human hematopoiesis (bone marrow) dataset, using a Leiden resolution of 1.0.

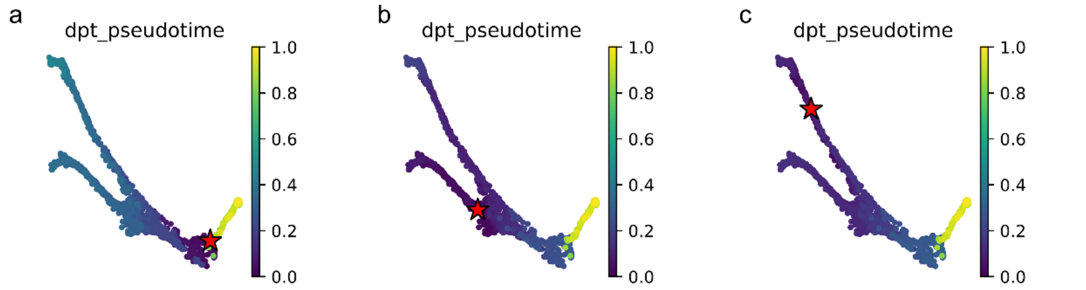

Figure S4. Visualization of Diffusion Pseudotime calculated from sub-optimal and incorrect root nodes in the zebrafish embryogenesis dataset.

These visualizations correspond to the root perturbation analysis presented in Table S1. a–c, Visualizations of the zebrafish embryogenesis dataset colored by Diffusion Pseudotime calculated using different root nodes: root1 (a), root2 (b), and root3 (c). In each panel, the red asterisk indicates the specific location of the selected root node used for the respective Diffusion Pseudotime calculation.

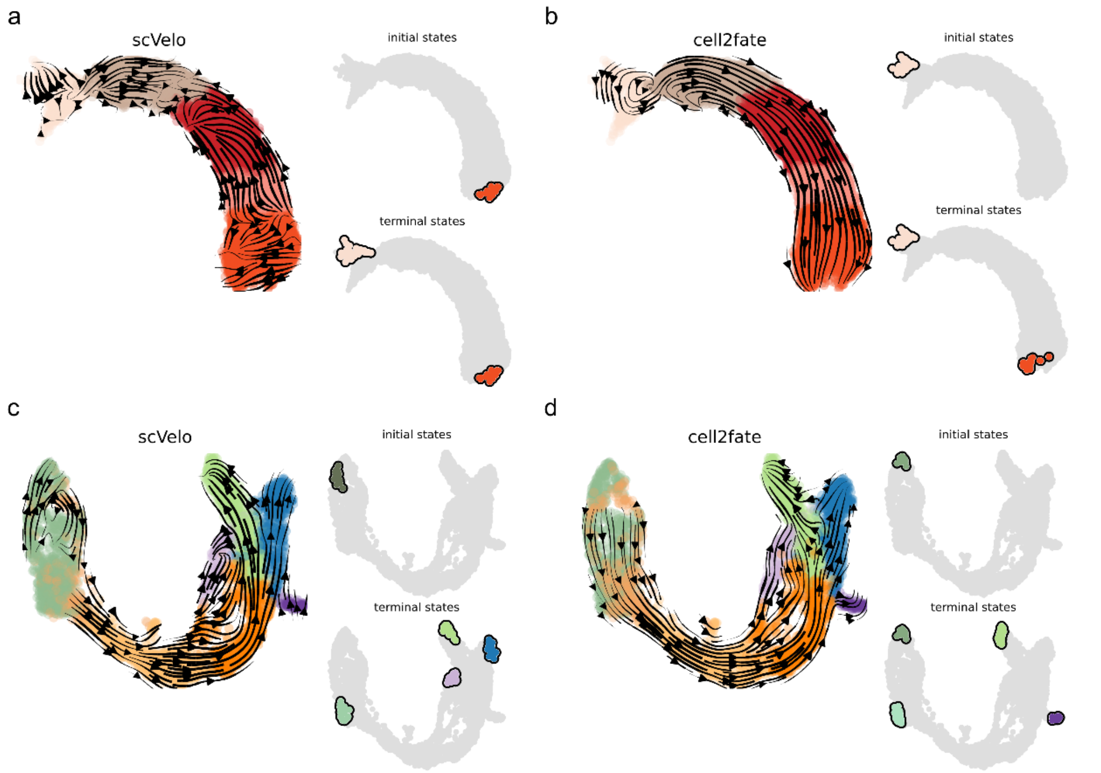

Figure S5. Streamline projections and identification of initial and terminal states by RNA velocity methods across representative developmental datasets.

These visualizations correspond to the quantitative benchmarking of traditional RNA velocity-based approaches (scVelo and Cell2fate) presented in Table S2. Initial and terminal states were identified using CellRank based on the prediction of scVelo and Cell2fate. a, b, Erythroid maturation dataset. Streamline projections and state identification based on the velocities derived from scVelo (a) and Cell2fate (b). c, d, Mouse pancreatic endocrinogenesis (Pancreas) dataset. Streamline projections and state identification based on the velocities derived from scVelo (c) and Cell2fate (d). For each panel (a–d), there are three sub-panels: the left sub-panel displays the streamline projections of the inferred developmental flows on the low-dimensional embeddings; the right two sub-panels display the biological cell fate predictions, with the top right sub-panel showing the identified initial states and the bottom right sub-panel showing the identified terminal states.

Table S1. Robustness benchmarking of PseudoVelo and CellRank 2 using sub-optimal and incorrect root nodes for Diffusion Pseudotime (DPT) calculation in the Zebrafish embryogenesis dataset.

Root	PseudoVelo			CellRank2		
	CBDC	LPC(median)	ITSA	CBDC	LPC(median)	ITSA
root1	0.78	0.82	0.75	0.55	0.25	0.75
root2	0.65	0.78	0.4	0.51	0.34	0.4
root3	0.57	0.68	0.4	0.5	0.34	0.4

To assess the impact of root node selection on model performance, Diffusion Pseudotime (DPT) was calculated using sub-optimal and incorrect root nodes (root1, root2, and root3) in the Zebrafish embryogenesis dataset and utilized as the temporal prior. Model robustness is quantitatively evaluated using three metrics: Cross-Boundary Direction Correctness (CBDC) for global developmental directionality, Local Projection Consistency (LPC, median) for the smoothness of the projected developmental flows, and Initial/Terminal State Accuracy (ITSA) for the biological relevance of cell fate prediction.

Table S2. Quantitative benchmarking of RNA velocity methods (scVelo and Cell2fate) across representative developmental datasets.

Dataset	scVelo			Cell2fate		
	CBDC	LPC(median)	ITSA	CBDC	LPC(median)	ITSA
Erythroid maturation	0.45	0.76	0.25	0.84	0.97	0.67
Pancreas	0.8	0.92	0.67	0.69	0.68	0.43

To provide a baseline comparison with traditional RNA velocity-based approaches, scVelo and Cell2fate were evaluated on the Erythroid maturation and Pancreas datasets. Model performance is quantitatively evaluated using three metrics: Cross-Boundary Direction Correctness (CBDC) for global developmental directionality, Local Projection Consistency (LPC, median) for the smoothness of the projected developmental flows, and Initial/Terminal State Accuracy (ITSA) for the biological relevance of cell fate prediction.

Table S3. Computational efficiency and memory usage of PseudoVelo across multiple developmental datasets.

Dataset	Number of Cells	Number of Genes	Runtime	Peak Memory
Erythroid maturation	9815	2000	442.88s	760.34MB
Zebrafish embryogenesis	2341	2000	76.44s	178.00MB
Pancreas	3686	2000	99.95s	207.38MB
Bone Marrow	5780	2000	409.32s	326.44MB

To assess the computational cost of PseudoVelo, the runtime and peak memory usage were evaluated across four representative developmental datasets (Erythroid maturation, Zebrafish embryogenesis, Pancreas, and Bone Marrow). The number of cells and the number of highly variable genes used for each dataset are provided to reflect the scale of the input data. All tests were performed on a workstation equipped with an Intel Core i7-10700K CPU and 32 GB of RAM, utilizing 10 parallel processes (n_jobs=10).
